# Validation of a portable three-dimensional imaging system for volumetric measurement in the periorbital region

**DOI:** 10.3389/fmed.2025.1647489

**Published:** 2025-08-08

**Authors:** Xuan Zhang, Ji Shao, Ningxin Dai, Huimin Li, Yongwei Guo, Juan Ye, Lixia Lou

**Affiliations:** Eye Center of the Second Affiliated Hospital, School of Medicine, Zhejiang University, Zhejiang Provincial Key Laboratory of Ophthalmology, Zhejiang Provincial Clinical Research Center for Eye Diseases, Zhejiang Provincial Engineering Institute on Eye Diseases, Hangzhou, China

**Keywords:** portable three-dimensional imaging system, structured light, volumetric measurement, reliability, accuracy

## Abstract

**Objectives:**

To evaluate the reliability and accuracy of a portable structured light-based three-dimensional (3D) imaging system for volumetric measurement in the periorbital region.

**Methods:**

Five hemispherical 3D printed resin models with different sizes and colors, including the flesh-colored models with 6 (Model 1), 4 (Model 2), and 2 (Model 3) mm in diameter, and the black (Model 4) and gray (Model 5) models with 6 mm in diameter, were affixed to the lower eyelid or upper eyelid of 40 volunteers. One researcher used the portable 3D imaging system to collect the 3D images and two raters measured the volume of the models on images. Intra-device, intra-rater and inter-rater reliabilities and accuracy of the volumetric measurement were evaluated by intraclass correlation coefficient (ICC), mean absolute difference (MAD), technical error of measurement (TEM), relative error measurement (REM), and relative TEM (rTEM).

**Results:**

The intra-device reliability of the 3D imaging system for volumetric measurement in the periorbital region was excellent (ICC = 0.922, MAD = 0.11 mm^3^, TEM = 0.09 mm^3^, REM = 0.19%, rTEM = 0.15%). The intra-rater reliability for volumetric measurement of the Model 1 on the lower eyelid was the highest (ICC = 0.953, MAD = 0.08 mm^3^, TEM = 0.06 mm^3^, REM = 0.13%, rTEM = 0.11%). The inter-rater reliability for volumetric measurement of the Model 1 on the lower eyelid was the highest (ICC = 0.889, MAD = 0.14 mm^3^, TEM = 0.11 mm^3^, REM = 0.24%, rTEM = 0.19%). The accuracy for volumetric measurement of the Model 1 on the lower eyelid was the highest (MAD = 0.24 mm^3^, REM = 0.43%).

**Conclusion:**

The portable 3D imaging system proved to measure the volumes of periorbital flesh-colored objects reliably and accurately. This finding demonstrated that this device has great potential for diagnosis, post-operative evaluation, and long-term follow-up of volumetric changes in oculoplastics.

## 1 Introduction

Oculoplastics, a subspecialty of ophthalmology and plastic surgery, focuses on treating the abnormalities of eye appearance (1), which can affect the overall appearance and life quality of the patients. Considering the high expectations of patients for treatment outcomes, accurate evaluations before and after treatment are beneficial for the communication between oculoplastic surgeons and patients. In recent years, facial anthropometric measurements, including direct anthropometry, cephalometry, two-dimensional (2D) digital photogrammetry and three-dimensional (3D) photogrammetry, have played an important role in disease diagnosis, clinical decision-making and prognostic follow-up (2–4). Compared to other methods of facial anthropometry, 3D photogrammetry offers the advantage of directly measuring the volume of a stereoscopic image, facilitating for a more comprehensive assessment of oculoplastic patients. Several studies have used 3D photogrammetry to quantify periorbital volume changes resulting from aging, upper eyelid blepharoplasty, lower eyelid blepharoplasty and filling material injections (5–9).

With the rise of 3D photogrammetry, 3D imaging systems based on different 3D imaging technologies, including stereophotogrammetry, laser scanning and structured light have been developed to meet different application scenarios (10–13). But before widely employed in clinical practice, the reliability and accuracy of any novel measurement technique must be fully validated. To date, the use of different 3D imaging systems to measure the volumes of the breast, arm, thigh, vulva, hand, and larger area of the face has been proven to be reliable (14–18). However, due to the small size of the periorbital region and the difficulty of data acquisition, only a few studies have validated the performance of 3D imaging systems for analyzing the volume in this region, thereby limiting their application (19). A previous study verified the reproducibility of a 3D imaging system employing stereophotogrammetry for measuring the volumetric of periorbital tumor, but did not assess accuracy (20). Another study evaluated the reliability of a static 3D imaging system and a portable 3D imaging system employing stereophotogrammetry for volume measurement in the upper eyelid region (21). Although static 3D imaging systems employing stereophotogrammetry have proven to be highly reliable, they are often costly and difficult to move, imposing significant limitations in practical clinical applications. Portable 3D imaging systems employing stereophotogrammetry, which generate 3D images by successively capturing photographs from three angles, are prone to motion artifacts and stitching errors when measuring the periorbital region, resulting in reduced accuracy.

By contrast, portable 3D imaging systems employing structured light are suitable for diverse working environments, such as bedside assessments and outreach clinics, and have the advantages of low cost, easy calibration and the ability to scan from multiple angles, but their ability for volumetric measurement in the periocular region has not yet been evaluated. Therefore, this study aimed to illustrate the reliability and accuracy of a portable 3D imaging system employing structured light for volumetric measurement in the periorbital region, including intra-device, intra-rater and inter-rater reliability.

## 2 Materials and methods

### 2.1 Participant population

Forty healthy volunteers aged from 22 to 29 years (mean ± SD, 25.1 ± 1.7 years) were recruited in this study, including 20 females (mean ± SD, 25.0 ± 1.7 years) and 20 males (mean ± SD, 25.3 ± 1.7 years). Exclusion criteria were individuals with a history of eyelid diseases (e.g., blepharoptosis, entropion, ectropion, enophthalmos, or exophthalmos), strabismus, abnormalities of pupil, injuries and plastic surgery or aesthetic procedures involving the periorbital area.

### 2.2 3D printed models and image acquisition

**Figure 1:**
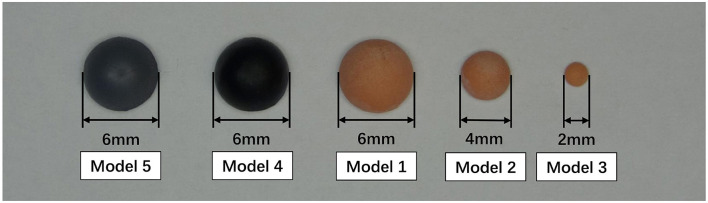
3D printed hemispherical resin models with different sizes and colors.

**Figure 2:**
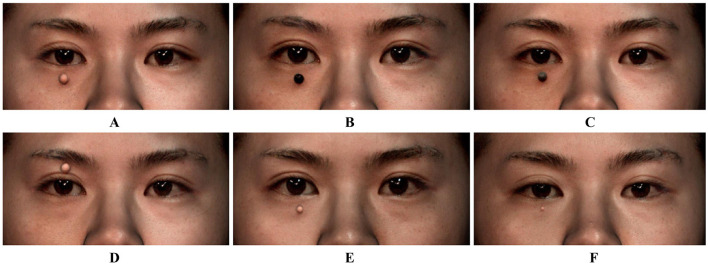
3D printed hemispherical resin models affixed to the periorbital region of volunteers. **(A)** Model 1 (6 mm, flesh-colored) placed on the lower eyelid and directly below the pupil; **(B)** Model 4 (6 mm, black) placed on the lower eyelid and directly below the pupil; **(C)** Model 5 (6 mm, gray) placed on the lower eyelid and directly below the pupil; **(D)** Model 1 (6 mm, flesh-colored) placed on the upper eyelid and directly above the pupil; **(E)** Model 2 (4 mm, flesh-colored) placed on the lower eyelid and directly below the pupil; **(F)** Model 3 (2 mm, flesh-colored) placed on the lower eyelid and directly below the pupil.

**Figure 3:**
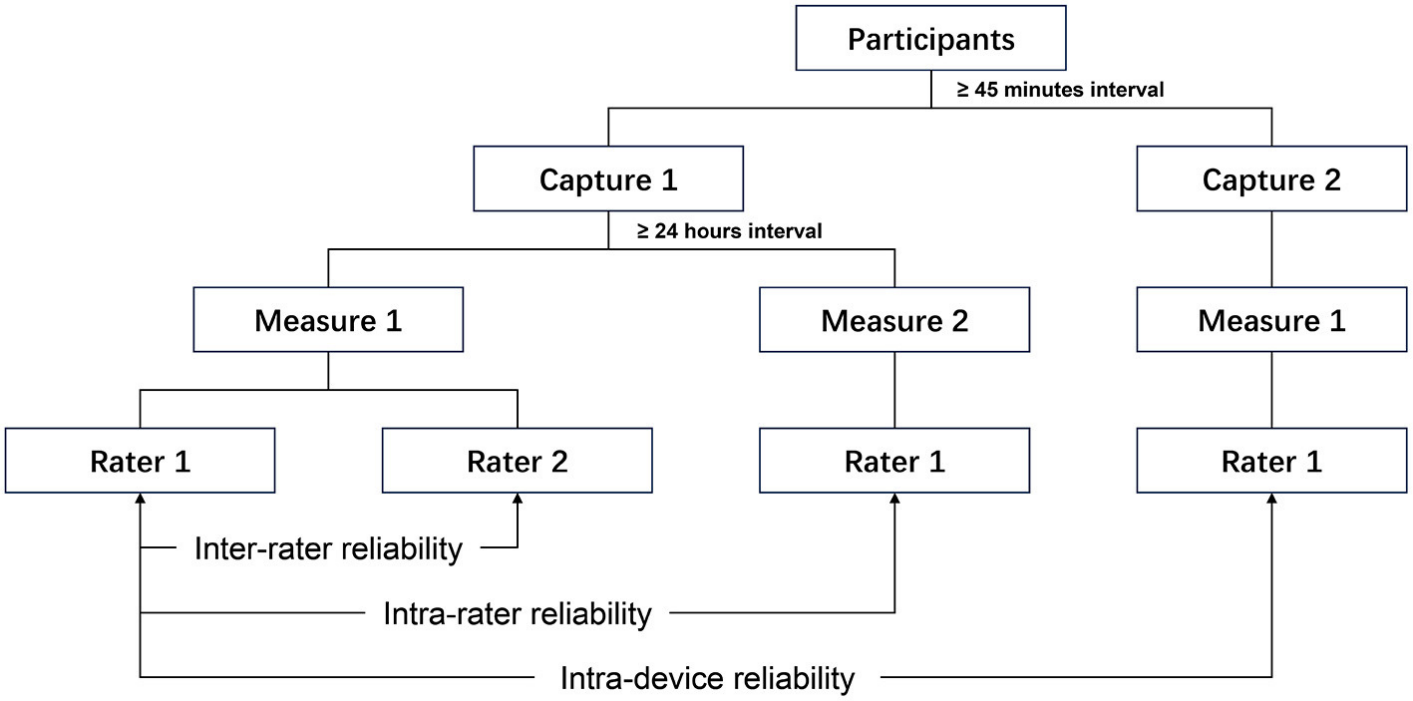
The data measurement strategy of verifying the reliability of the portable 3D imaging system.

Five types of hemispherical resin models with different sizes and colors are printed by 3D printers, including the flesh-colored models of 6, 4, and 2 mm in diameter, and the black and gray models of 6 mm in diameter, which were numbered 1 to 5, respectively (Figure 1). All volunteers were requested to remove makeup and to pull their hair back to fully expose their forehead and eyebrows. An experienced researcher (X.Z.) affixed these models to the periorbital region of the volunteers for volumetric measurement and collected 3D images using the portable structured light-based 3D imaging system iReal 2E (SCANTECH, Hangzhou, China). The Model 1 to 5 were placed sequentially on the lower eyelid and directly below the pupil. Besides, the Model 1 was also placed on the upper eyelid and directly above the pupil. All models were placed at a distance of 5 mm from the eyelid margins (Figure 2). During the image capture process, to minimize errors caused by involuntary head movements, facial expression changes, and frequent blinking, volunteers were asked to sit in a chair with their backs resting against it, hold their heads straight, and relax their facial muscles, while gazing at a stationary light source two meters straight ahead and consciously avoiding blinking for at least 5 s. To test the reproducibility of portable 3D imaging system for volumetric measurement, volunteers with Model 1 placed on the lower eyelid were scanned a second time by the same researcher, after at least 45 minu of the first scan (Capture 1 and Capture 2 in Figure 3). Image acquisitions were conducted in the same clinical photography room under the same lighting conditions.

### 2.3 Data measurements

To assess the inter-rater reliability, two independent raters [Rater 1 (X.Z.) and Rater 2 (J.S.) in Figure 3] performed the volumetric measurements of models using GOM Inspect 2019 (GOM GmbH, Braunschweig, Germany). To assess the intra-rater reliability, the Rater 1 repeated the measurements twice for each image (Measure 1 and Measure 2 in Figure 3) with a minimum 24-h interval.

### 2.4 Statistical analysis

Five statistics were calculated to assess the reliability, including intraclass correlation coefficient (ICC), mean absolute difference (MAD), technical error of measurement (TEM), relative error measurement (REM), and relative TEM (rTEM). The formulas for these statistics are as follows:


$$\begin{array}{r} MAD=\sum D\\ REM=\sum \frac{D}{M}\times 100\%\\ TEM=\sqrt{(\sum D^{2})/2N}\\ rTEM=\frac{TEM}{mean}\times 100\% \end{array}$$

where D represents the difference between the two measurements, N represents the number of individuals measured, M represents Measure 1 of Rater 1 in Figure 3, and mean represents the average of all measurements. For ICC, < 0.5, 0.5–0.75, 0.75–0.90 and ≥0.90 were considered poor, moderate, good, and excellent agreement, respectively (22). For MAD and TEM, the acceptable error thresholds were set to <1 unit (23). For REM and rTEM, < 1%, 1–3.9%, 4–6.9%, 7–9.9%, and ≥10% were deemed excellent, very good, good, moderate, and poor agreement, respectively (24). Heat maps were constructed using GraphPad Prism 8 (GraphPad Software Inc., San Diego, USA). Statistical analyses were performed using SPSS 27 (IBM Corp., Armonk, USA). For normally distributed measurement data, the paired-samples *t*-test was used to assess intra-device, intra-rater, and inter-rater reliabilities, while the non-parametric Wilcoxon signed-rank test for paired data was applied for non-normally distributed measurement data. The level of statistical significance was set at *p* < 0.05.

## 3 Results

### 3.1 Intra-device reliability of portable 3D imaging system for volumetric measurement

On 3D images acquired from two captures, the volume of Model 1 (6 mm, flesh-colored) affixed to the lower eyelid were 56.37 ± 0.30 mm^3^ and 56.36 ± 0.32 mm^3^, respectively, and there was no statistically significant difference between these two measurements (*p* = 0.605). For the intra-device comparison, ICC, MAD, TEM, REM and rTEM were 0.922, 0.11 mm^3^, 0.09 mm^3^, 0.19% and 0.15%, respectively, which were clinically acceptable errors and demonstrated excellent agreement.

### 3.2 Intra-rater reliability of portable 3D imaging system for volumetric measurement

**Figure 4:**
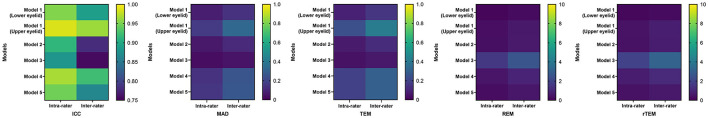
Heat maps of the reliability of the portable 3D imaging system for volumetric measurement.

The ICC estimates for the intra-rater comparison were all more than 0.90 (Table 1, Figure 4), which displayed excellent agreement, except the ICC of Model 3 (2 mm, flesh-colored) (ICC = 0.880). The ICC of Model 1 (6 mm, flesh-colored) affixed to the upper eyelid was the highest (ICC = 0.986).

**Table 1:** Intra-rater reliability of the portable 3D imaging system for volumetric measurement.

**Models**	**Measure 1 (mm** ^**3**^ **)**		**Measure 2 (mm** ^**3**^ **)**		**ICC**	**MAD (mm^3^)**	**TEM (mm^3^)**	**REM (%)**	**rTEM (%)**	***p*-value**
	**Mean**	**SD**	**Mean**	**SD**						
Model 1 (lower eyelid)	56.37	0.30	56.36	0.28	0.953	0.08	0.06	0.13	0.11	0.176
Model 1 (upper eyelid)	51.91	1.45	51.87	1.46	0.986	0.17	0.17	0.33	0.33	0.428
Model 2	16.55	0.17	16.56	0.18	0.913	0.06	0.05	0.37	0.30	0.604
Model 3	2.09	0.09	2.09	0.08	0.880	0.03	0.03	1.60	1.37	0.937
Model 4	26.80	0.76	26.78	0.71	0.967	0.14	0.13	0.52	0.50	0.396
Model 5	46.76	0.55	46.80	0.60	0.946	0.15	0.13	0.31	0.28	0.217

For all models, the MAD and TEM were <1 unit (Table 1, Figure 4), which were clinically acceptable errors. For models with different sizes, the MAD and TEM of Model 3 (2 mm, flesh-colored) were the smallest (MAD = 0.03 mm^3^, TEM = 0.03 mm^3^). For models with different colors, the MAD and TEM of Model 1 (6 mm, flesh-colored) were the smallest (MAD = 0.08 mm^3^, TEM = 0.06 mm^3^). For models with different positions, the MAD and TEM of Model 1 affixed to the lower eyelid were smaller than these of Model 1 affixed to the upper eyelid (MAD = 0.17 mm^3^, TEM = 0.17 mm^3^).

For all models, the REM and rTEM were < 4% (Table 1, Figure 4), which displayed very good agreement. For models with different sizes, the REM and rTEM of Model 1 (6 mm, flesh-colored) were the smallest (REM = 0.13%, TEM = 0.11%). For models with different colors, the REM and rTEM of Model 1 were still the smallest. For models with different positions, the REM and rTEM of Model 1 affixed to the lower eyelid were smaller than these of Model 1 affixed to the upper eyelid (REM = 0.33%, TEM = 0.33%).

In the intra-rater comparison of two volumetric measurements performed by Rater 1, there were no statistically significant difference (*p* > 0.05) (Table 1).

### 3.3 Inter-rater reliability of portable 3D imaging system for volumetric measurement

The ICC estimates for the intra-rater comparison were all more than 0.75 (Table 2, Figure 4), which indicated good agreement. The ICC of Model 1 (6 mm, flesh-colored) affixed to the upper eyelid was the highest (ICC = 0.963).

**Table 2:** Inter-rater reliability of the portable 3D imaging system for volumetric measurement.

**Models**	**Rater 1 (mm** ^**3**^ **)**		**Rater 2 (mm** ^**3**^ **)**		**ICC**	**MAD (mm^3^)**	**TEM (mm^3^)**	**REM (%)**	**rTEM (%)**	***p*-value**
	**Mean**	**SD**	**Mean**	**SD**						
Model 1 (lower eyelid)	56.37	0.30	56.40	0.34	0.889	0.14	0.11	0.24	0.19	0.340
Model 1 (upper eyelid)	51.91	1.45	51.94	1.60	0.963	0.33	0.29	0.63	0.56	0.605
Model 2	16.55	0.17	16.57	0.19	0.780	0.11	0.08	0.66	0.51	0.285
Model 3	2.09	0.09	2.09	0.10	0.756	0.05	0.05	2.59	2.19	0.939
Model 4	26.80	0.76	26.77	0.74	0.923	0.26	0.21	0.96	0.78	0.538
Model 5	46.76	0.55	46.81	0.60	0.865	0.27	0.21	0.57	0.46	0.310

For all models, the MAD and TEM were <1 unit (Table 2, Figure 4), which were clinically acceptable errors. For models with different sizes, the MAD and TEM of Model 3 (2 mm, flesh-colored) were the smallest (MAD = 0.05 mm^3^, TEM = 0.05 mm^3^). For models with different colors, the MAD and TEM of Model 1 (6 mm, flesh-colored) were the smallest (MAD = 0.14 mm^3^, TEM = 0.11 mm^3^). For models with different positions, the MAD and TEM of Model 1 affixed to the lower eyelid were smaller than these of Model 1 affixed to the upper eyelid (MAD = 0.33 mm^3^, TEM = 0.29 mm^3^).

For all models, the REM and rTEM were < 4% (Table 2, Figure 4), which indicated very good agreement. For models with different sizes, the REM and rTEM of Model 1 (6 mm, flesh-colored) were the smallest (REM = 0.24%, TEM = 0.19%). For models with different colors, the REM and rTEM of Model 1 were still the smallest. For models with different positions, the REM and rTEM of Model 1 affixed to the lower eyelid were smaller than these of Model 1 affixed to the upper eyelid (REM = 0.63%, TEM = 0.56%).

In the inter-rater comparison of volumetric measurements performed by Rater 1 and Rater 2, there were no statistically significant difference (*p* > 0.05) (Table 2).

### 3.4 Accuracy of portable 3D imaging system for volumetric measurement

For models with different sizes, the true volumes of Model 1 (6 mm, flesh-colored), Model 2 (4 mm, flesh-colored) and Model 3 (2 mm, flesh-colored) were 56.55 mm^3^, 16.76 mm^3^, and 2.09 mm^3^. The volumetric measurements performed by Rater 1 for Model 1, Model 2, and Model 3 affixed to the lower eyelid were 56.37 ± 0.30 mm^3^, 16.55 ± 0.17 mm^3^, and 2.09 ± 0.09 mm^3^. In the comparison between true volumes and volumetric measurements, the MAD of Model 1, Model 2 and Model 3 were 0.24 mm^3^, 0.20 mm^3^, and 0.07 mm^3^, which were all clinically acceptable errors, while the REM of Model 1, Model 2 and Model 3 were 0.43%, 1.22% and 3.21%, which revealed excellent, very good and very good accuracy, respectively (Table 3).

**Table 3:** Accuracy of the portable 3D imaging system for volumetric measurement.

**Models**	**Rater 1 (mm** ^**3**^ **)**		**True volume (mm^3^)**	**MAD (mm^3^)**	**REM (%)**
	**Mean**	**SD**			
Model 1 (lower eyelid)	56.37	0.30	56.55	0.24	0.43
Model 1 (upper eyelid)	51.91	1.45	56.55	4.64	8.21
Model 2	16.55	0.17	16.76	0.20	1.22
Model 3	2.09	0.09	2.09	0.07	3.21
Model 4	26.80	0.76	56.55	29.75	52.61
Model 5	46.76	0.55	56.55	9.79	17.31

For models with different colors, the true volumes of Model 4 (6 mm, black) and Model 5 (6 mm, gray) were the same as the true volume of Model 1 (6 mm, flesh-colored), which were 56.55 mm^3^. The volumetric measurements performed by Rater 1 for Model 1, Model 4 and Model 5 were 56.37 ± 0.30 mm^3^, 26.80 ± 0.76 mm^3^, and 46.76 ± 0.55 mm^3^. In the comparison between true volumes and volumetric measurements, the MAD of Model 1, Model 4 and Model 5 were 0.24 mm^3^, 29.75 mm^3^, and 9.79 mm^3^, while the REM of Model 1, Model 4, and Model 5 were 0.43%, 52.61%, and 17.31% (Table 3).

For models with different positions, the true volume of Model 1 (6 mm, flesh-colored) affixed to either the lower or upper eyelid was 56.55 mm^3^. The volumetric measurements performed by Rater 1 for Model 1 affixed to the lower eyelid and upper eyelid were 56.37 ± 0.30 mm^3^ and 51.91 ± 1.45 mm^3^. In the comparison between true volumes and volumetric measurements, the MAD of Model 1 affixed to the lower eyelid and upper eyelid were 0.24 mm^3^ and 4.64 mm^3^, while the REM of Model 1 affixed to the lower eyelid and upper eyelid were 0.43% and 8.21%, which revealed excellent and moderate accuracy, respectively (Table 3).

## 4 Discussion

In this study, we conducted a comprehensive evaluation of the ability of a portable structured light-based 3D imaging system for measuring the volumes of resin models affixed to the periorbital region. For volumetric measurements of models with different sizes, colors and positions, excellent intra-device, intra-rater and inter-rater reliability and accuracy were detected in the volumetric measurement of the largest flesh-colored model affixed to the lower eyelid. As a supplement to previous research on validating the reliability and accuracy of 3D imaging systems, this study provided support for the application of the portable structured light-based 3D imaging systems to volumetric measurement in the periorbital region. Our findings extended the clinical applications of the 3D imaging system, which has the potential to be a new technique for diagnosis, post-operative evaluation and long-term follow-up of volume changes in oculoplastics. This advancement lays the foundation for researchers to integrate it with artificial intelligence technology in developing automatic 3D diagnostic and therapeutic models in the future.

For intra-device reliability, the volumetric measurement of Model 1 (6 mm, flesh-colored) affixed to the lower eyelid demonstrated excellent agreement in all statistics. In 2022, Fan et al. pioneered an assessment of the reliability of the static 3D imaging system VECTRA M3 for volumetric measurement by indirectly measuring the volume of periorbital tumor models, but did not evaluate the intra-device reliability (20). Shortly afterwards, a study by Fan et al. in 2023 detected that the static VECTRA M3 and portable VECTRA H2 showed moderate and poor intra-device reliability in measuring the upper eyelid region volume (21). The results of our study illustrated that the portable structured light-based 3D imaging system iReal 2E exhibited high intra-device reliability in measuring large flesh-colored hemispherical models located on the lower eyelid. This was likely because the imaging process of this device relied on active projection of infrared light and continuous scanning, where the reproducibility of repeated scans was less affected by variations in ambient light and image stitching errors. Future studies could further validate the intra-device reliability of this portable 3D imaging system for measuring the volume of periorbital models with different sizes, shapes, colors, and locations.

Within models with different sizes, the intra-rater reliability, inter-rater reliability and accuracy of the volumetric measurement for Model 1 (6 mm, flesh-colored) were the highest. Although the MAD and TEM of Model 3 (2 mm, flesh-colored) was the smallest, the REM and rTEM were more reflective of the level of reliability and accuracy due to the large differences in volume of models with different sizes. Our results suggested that the ability of this portable 3D imaging system for volumetric measurement in the periorbital region increased as the volume of the measurement object increased, which was consistent with previous studies. However, compared to the study by Fan et al. (20), this device demonstrated higher reliability in volumetric measurements of models with the same size. This was attributed to its capability of allowing researchers to perform scans from multiple angles, thereby enabling more accurate capture of subtle volume changes. Evaluating periorbital tumors was one of the clinical applications of 3D photogrammetry that researchers were looking forward to. Studies have shown that the optimal treatment of periorbital tumors depends on tumor type and size, and a diameter >6 mm is considered one of the features associated with malignant lesions (25, 26). Therefore, our results demonstrated that this portable 3D imaging system could be used to aid in early identification and treatment decisions for malignant periorbital tumors.

Within models with different colors, the volumetric measurement for Model 1 (6 mm, flesh-colored) was the most reliable and accurate, followed by Model 5 (6 mm, gray), while that of Model 4 (6 mm, black) was poor. These results demonstrated that this portable 3D imaging system performed better when measuring the volume of flesh-colored objects in the periorbital region, which could be explained by the imaging principles. Structured light technology generated spatial coordinates and rendered 3D models through projecting a known light pattern onto the measurement object and capturing the light reflected from it by cameras from different angles with known deviations (27). Because of the low reflectivity of black surfaces, black models in the periorbital region were poorly modeled, which affected the volumetric measurement. Therefore, with limited application in accurately measuring the volumes of black lesions, this structured light-based 3D imaging system was more suitable for measuring the volumes of flesh-colored tumors in the periorbital region, such as basal cell carcinoma, the most common malignant eyelid tumor (26, 28), as well as the volume changes resulting from aging or eyelid blepharoplasty.

Within models with different positions, the reliability and accuracy of the volumetric measurement of Model 1 (6 mm, flesh-colored) affixed to the lower eyelid were higher than that of Model 1 affixed to the upper eyelid. These results demonstrated that this 3D imaging system performed better in measuring lower eyelid volume, which might be explained by the fact that lower eyelid region was flatter than the upper eyelid, which contributed to the accuracy of measurements. Considering the difficulty in estimating the amount of fat removed during lower blepharoplasty and the fact that some periorbital tumors preferentially occur in the lower eyelid, this 3D imaging system still had a promising clinical application (29, 30). Besides, It is interesting that the reliability of iReal 2E for measuring upper eyelid volume was higher than that of VECTRA M3 and VECTRA H2 (21). The reasons include the fact that this study used the direct method rather than the superimposed method to measure the volume, and this 3D imaging system based on structured light could scan from more angles, which enabled the capture of irregular surfaces more accurately (31).

### 4.1 Limitation

There are some limitations should be noticed in this study. Firstly, the portability of the device should be proven in more scenarios with different lighting conditions. Secondly, all volunteers recruited in this study were healthy Asians. More geographically and ethnically diverse volunteers need to be included for comprehensive validation. Thirdly, as this study measured the volume of models to validate the portable 3D imaging system for volumetric measurement, further studies are required to evaluate the usability of this device in clinical studies, such as measuring the eyelid tumor volume and volume changes before and after oculoplastic surgery.

## 5 Conclusion

This study evaluated the reliability and accuracy of a portable 3D imaging system for volumetric measurement of hemispherical 3D printed resin models affixed to the periorbital region. Through the analysis of intra-device, intra-rater and inter-rater reliabilities, as well as a comparison of measured vs. true values, the study demonstrated the appropriateness of this device for measuring the volumes of flesh-colored models. These findings indicate that this device holds great potential for accurately measuring volume changes in the periorbital region, thus providing valuable assistance in oculoplastic surgeries.

## Data Availability

The raw data supporting the conclusions of this article will be made available by the authors, without undue reservation.
